# Evaluation of the Pharmaceutical Quality of Docetaxel Injection Using New Stability Indicating Chromatographic Methods for Assay and Impurities

**DOI:** 10.3797/scipharm.0912-14

**Published:** 2010-03-05

**Authors:** Annarapu Malleswara Reddy, Nagaraju Banda, Shinde Govind Dagdu, Dama Venugopala Rao, Chandra Sekhar Kocherlakota, Vyas Krishnamurthy

**Affiliations:** 1 Analytical Research and Development, Integrated Product Development, Dr. Reddy’s Laboratories Ltd., Bachupally, Hyderabad-500 072, India; 2 Project Management, Integrated Product Development, Dr. Reddy’s Laboratories Ltd., Bachupally, Hyderabad-500 072, India

**Keywords:** HPLC, Quality, Stability indicating, Docetere, Impurities, Excipients

## Abstract

New stability indicating chromatographic methods have been developed for estimation of Assay and Impurities of Docetaxel in Docetaxel injection for evaluation of pharmaceutical quality. With this method, the process related impurities and degradants are well separated from the peaks due to placebo. The relative retention times and relative response factors of the known impurities have been established. The LOQ of the known impurities and docetaxel are found to be less than 0.2 μg /ml and the recovery falls in the range of 90–110%. Peak purities demonstrated the stability indicating nature of the methods. The methods developed in the present study overcome the lacunae of the existing published methodologies in evaluation of the quality of Docetaxel injection. In essence, the present study provides an improved methodology for evaluation of the pharmaceutical quality of Docetaxel injection.

## Introduction

**Fig. 1.**
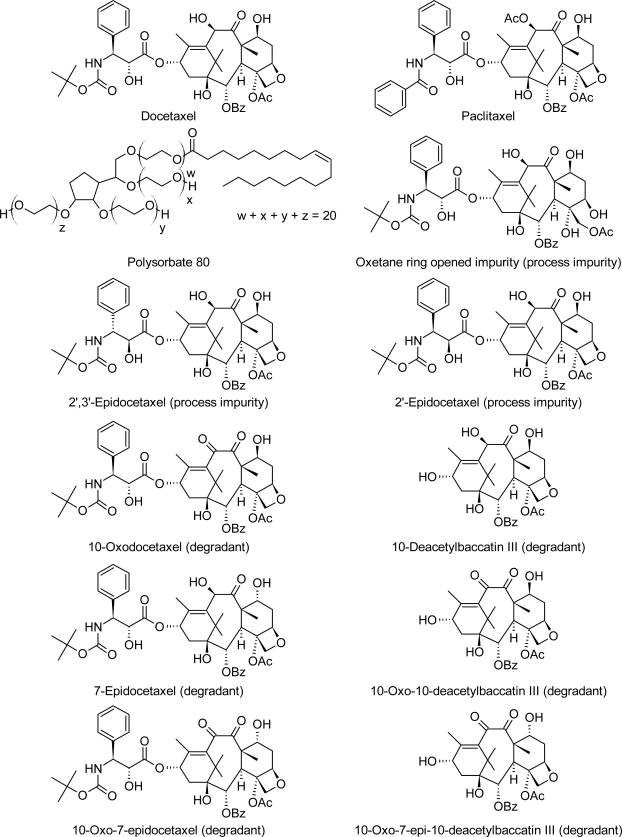
Structures of Docetaxel, Paclitaxel, Polysorbate 80 and Impurities of Docetaxel.

Docetaxel is an antineoplastic agent and belongs to the taxoid family. Docetaxel is of the chemotherapy drug class of taxanes and is a semi-synthetic analogue of paclitaxel, an extract from the rare Pacific yew tree Taxus brevifolia. Due to scarcity of paclitaxel, extensive research was carried out leading to the discovery of docetaxel – an esterified product of 10-deacetyl baccatin III, which is extracted from the renewable and readily available European yew tree. Docetaxel differs from Paclitaxel at two positions in its chemical structure (Figure 1). It has a hydroxyl functional group on carbon 10, whereas paclitaxel has an acetate ester and a *tert*-butyl substitution exists on the phenyl propionate side chain. Docetaxel is a white powder.

Docetaxel formulation is supplied as injection concentrate as 20 mg and 80 mg single-dose vials as a sterile, pyrogen-free, non-aqueous, viscous solution by Sanofi-Aventis under brand name of Taxotere^®^. A sterile, non-pyrogenic, single-dose diluents (13% ethanol in water for injection) is supplied for dilution of the formulation [1, 2]. Docetere™ is supplied as 20 mg and 80 mg single-dose vials as a sterile, pyrogen-free, non-aqueous, viscous solution by Dr. Reddy’s Laboratories Limited along with a sterile, non-pyrogenic, single-dose diluents (13% ethanol in water for injection) for dilution of the formulation.

The evaluation of quality of a drug substance requires complete understanding on the chemistry of drug molecule, its potential process and degradation related impurities. Isolation and characterization of some process related impurities [3] and degradation impurities of docetaxel are also published [4]. A stability indicating HPLC assay method for Docetaxel has been reported [5]. A method for estimation of related substances of Docetaxel trihydrate drug substance is also published in Pharmeuropa [6]. The methods for the determination of Docetaxel in human plasma are available in literature [7–9].

On the other hand, the evaluation of quality of a drug product requires complete understanding of the placebo matrix used for formulation and degradation of the drug in presence of the placebo during storage throughout its shelf life. Methods for determination of docetaxel and its related substances in docetaxel injection by HPLC are also reported [10, 11], but no details of degradants of docetaxel in the docetaxel formulation are given. Jerome et al [12] have made an attempt to determine the pharmaceutical quality of docetaxel formulations, by employing a method for limit of degradation products of paclitaxel injection given in United States Pharmacopeia (USP).

In essence, the methods in the literature address impurities which are either process related or degradation related. There are no attempts made to separate and identify the drug related impurities in presence of formulation matrix. According to the current good manufacturing practices, all drug products must be tested with stability indicating methods wherein all the impurities are separated from placebo. Attempts are required to systematically understand appropriate and adequate methodologies of evaluation of quality of drug product of docetaxel injection. Hence the present study is aimed at developing a new stability indicating chromatographic method for estimation of docetaxel and its degradation products in docetaxel injection. The present work establishes methodology for the evaluation of docetaxel injection involving accurate identification and quantification Drug related impurities in Docetaxel injection by overcoming the lacunae existing in the published methodologies.

## Results and Discussion

### Impurities

Initially, attempts were made to understand the docetaxel formulation matrix before evaluation of the formulation’s quality. Doecetaxel is practically insoluble in water and freely soluble in anhydrous ethanol and soluble in methylene chloride [6]. Due to its insoluble nature, polysorbate 80 is used as a solubilizer to formulate docetaxel as injection. The innovator product Taxotere^®^ is a formulation of docetaxel using polysorbate 80 [1, 2]. Most generic formulations of docetaxel injection also contain polysorbate 80. Therefore, the present study focuses only on the estimation of impurities in docetaxel formulation with polysorbate 80 as matrix.

The polysorbate 80 is a nonionic surfactant and emulsifier derived from polyethoxylated sorbitan and oleic acid, which comes from fruit and berries and is often used in foods. In the nomenclature of polysorbates, the numeric designation following polysorbate refers to the lipophilic group, in this case the oleic acid. Polysorbate 80 has been widely used as emulsifier and solubilizer in Pharmaceutical. Polysorbate 80 is official in NF (USP), Japanese Pharmacopoeia (JP) and European Pharmacopoeia (Ph. Eur.). The quality of the Polysorbate 80 is evaluated by the tests like specific gravity, viscosity, acid value, hydroxyl value, saponification value, water content, residue on ignition, heavy metals and organic volatile impurities apart from identification tests.

Due to its chemical structure (Figure 1), polysorbate 80 gives peaks in chromatographic methods, depending on the detector that is employed. Hence, to evaluate any formulation that contains polysorbate 80 one needs to study the interference of the peaks due to polysorbate 80 for the estimation of drug related impurities. Therefore, the separation of drug related impurities from the peaks due to polysorbate 80 is essential in making the quality assessment of the docetaxel injection. Hence, the knowledge of degradation products of docetaxel plays a key role in evaluation of pharmaceutical quality of docetaxel injection. The structures of potential process related impurities of docetaxel trihydrate drug substance and the degradation products of docetaxel are listed in Figure 1.

An adequate methodology for assessing the quality of docetaxel injection needs to address the separation of potential process related impurities & degradants from each other and from placebo peaks if any. Otherwise, the methods would suffer from the following disadvantages. (a) Lack of clarity regarding the peaks due to impurities and placebo would lead to possibility of estimation of placebo peaks as Impurities of docetaxel. (b) In cases where peaks due to impurities and placebo merge, the impurities peaks likely to get excluded as placebo, leading to possibility underestimation of impurities (c) In cases where the placebo components interfere with drug peak and impurity peaks will lead to possible overestimation. None of the publications available in open literature made any mention about efforts to specifically separate the degradation products from placebo components and assess the quality by its drug related impurities. Hence these methods are not adequate for estimation of drug related impurities in docetaxel injection.

**Fig. 2.**
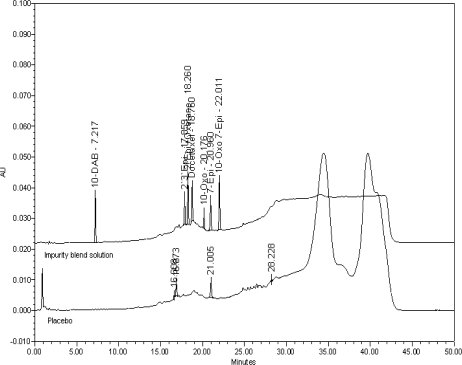
Overlay chromatogram of placebo with impurities blend (0.6%) in the literature method [12].

The recent publication [12] makes no mention of details to differentiate the drug related impurities from placebo peaks. On employing this method, it is found to be deficient for analysis of docetaxel formulation on the following two counts. Firstly, as a major degradant (7-epi Docetaxel) is found to be merging with placebo peak at relative retention time of 1.14. The overlay chromatogram of placebo with impurities at 0.6% level is shown in Figure 2. Secondly, other placebo peaks at relative retention time of 0.88, 0.90 and 1.51 are considered as impurities of docetaxel. The following points are considered as mandatory in the present study to finalize the analytical method as suitable in evaluation of the quality of the formulation in addition to the method being stability indicating. (a) Determination of retention times of the drug related impurities authentically. (b) Establishment of relative response factors to facilitate accurate quantification of known impurities. (c) Verification of the sensitivity of the analytical method by establishing the LOQ of the drug and its known impurities. All the above points have not been addressed in the published methods, related to docetaxel formulation, known till date.

**Fig. 3.**
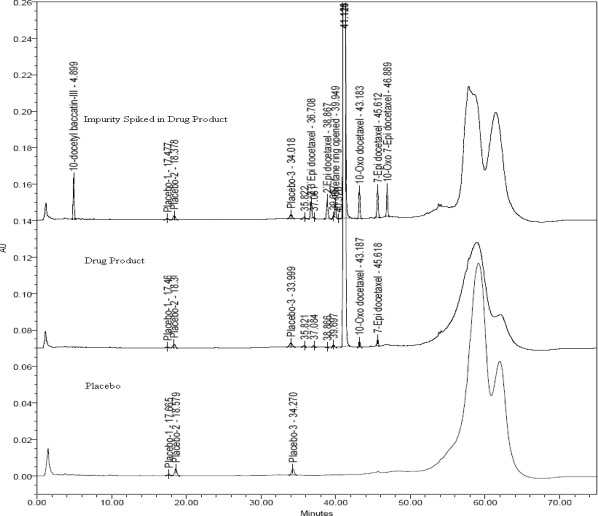
Overlay of chromatograms of Placebo (bottom), drug product (middle) and impurities spiked drug product (top).

In the present study, all the above requirements have been met leading to right methodology involving accurate identification and quantification drug related impurities in docetaxel formulations. The new chromatographic method developed is found to be capable of separating the process impurities, degradation products of docetaxel from placebo. The overlay chromatogram of placebo, drug product as such and drug product spiked with impurities is shown in Figure 3.

**Fig. 4.**
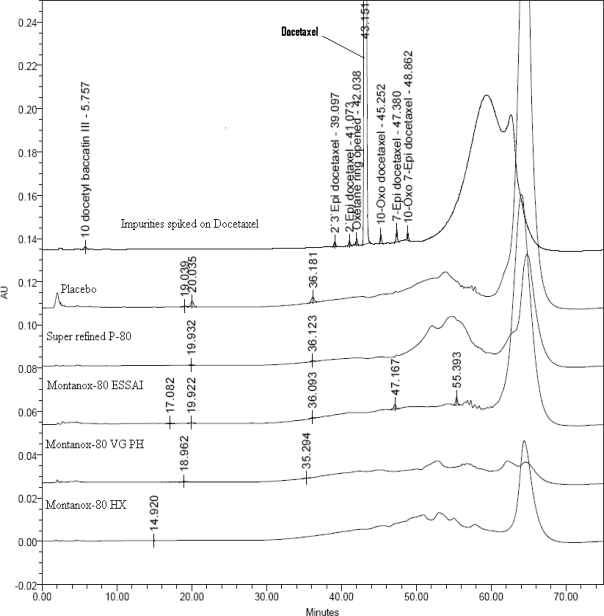
Overlay of chromatograms of impurities spiked drug product and different grades of polysorbate 80.

The authentic impurity standards are used for establishing their relative retention times. As standards of 7-epi-10-deacetylbaccatin III, 7-epi-10-oxo-10-deacetylbaccatin III are not available, the retention times are established based on forced degradation followed by LCMS study. The method has also been proved to be suitable for separation of drug related impurities from peaks of different grades of polysorbate 80 (Figure 4). However, as different docetaxel injection formulations can use different placebo components, it is appropriate to separate peaks due to placebo from the drug related impurities while evaluation of quality of docetaxel formulations.

### Method development and optimization

The HPLC method is optimized with a view to develop a stability indicating Assay and impurities methods. 7-epi Docetaxel, a major degradation product is found to be merging with placebo peak at relative retention time of 1.14 in the literature methods. The key objective of ‘chromatographic method’ is to get the separation of 7-epidocetaxel from placebo peak. Placebo and pure drug along with its related impurities are injected and run in different solvent systems. Our preliminary trials using different compositions of water, acetonitrile on different reversed phase stationary phases (ACE and X-Terra columns) did not give separation between pacebo peak and 7-epidocetaxel in a C-18 column. Introduction of another organic modifier methanol into the mobile phase resulted in moving the placebo peak away from impurities. Then the gradient program is optimized to elute the early eluting degradants like 10-DAB and to get the adequate separation of placebo peaks from all impurities in a C-18 Column.

### Specificity and mass balance study

**Fig. 5.**
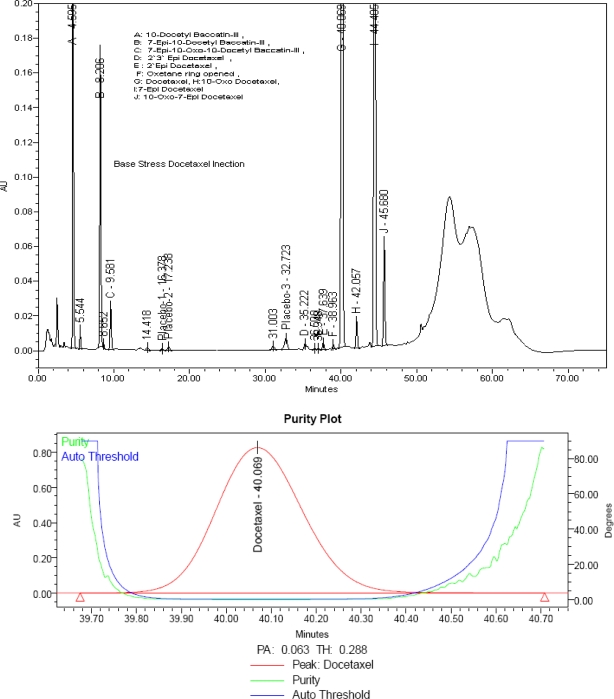
Chromatogram and purity plot of base stressed docetaxel injection

No considerable degradation is observed in docetaxel drug product under stress conditions of photolytic stress. Stress study conditions and mass balance data are given in Table 1. Peak purity test results obtained from PDA confirm that the docetaxel peak is homogeneous and pure in all the analyzed stress samples. The mass balance data of stressed samples is greater than 96%, which confirms the stability indicating power of the method. The base stressed chromatogram and purity plot of docetaxel injection is shown in Figure 5.

### Relative response factors

Relative response factors (RRF) are established for all the known impurities as the ratio of slope of impurities and slope of docetaxel. Slope value obtained with linearity calibration plot is used for RRF determination. Established RRF values are shown in Table 2.

### Precision

The precision of the method is evaluated by analyzing six samples of test preparation of docetaxel injection spiked with Impurities at 0.3% level. The Relative standard deviation is found to be less than 5.0% for all the impurities.

### Limit of detection (LOD) and limit of quantification (LOQ)

The limit of detection, limit of quantification and precision at LOQ values for docetaxel and its impurities are shown in Table 2. The precision at LOQ for all the impurities and docetaxel is in the RSD range of 1.5–3.7%.The recovery at LOQ level for the impurities is in the range of 96.1–108.3%.

### Accuracy

Recovery study of the impurities are performed at 0.15%, 0.3% and 0.45% levels and found that accuracy of the method falls in the range of 90.0% to 110.0%. Accuracy data is shown in Table-3. In the new method, care was taken to ensure adequate separation of drug related impurities from peaks due to placebo leading to accurate quantification of the impurities. The formulation samples of Docetere™ (Dr Reddy’s product) and Taxotere® (Innovator product) were analyzed. The % of impurities are corrected for their differences in responses in chromatography. The results are shown in Table 4

### Linearity

Linear calibration plot for docetaxel and its impurities is established over the concentration range of LOQ to 0.45%. The correlation coefficients are found to be greater than 0.998. This shows that an excellent correlation existed between the peak area and the concentration of impurities.

### Robustness

In all the deliberately altered chromatographic conditions (flow rate, column temperature and composition of organic solvent), all impurities are adequately resolved and elution orders remained unchanged. The resolution between critical pair, i.e. 2′-epidocetaxel and the oxetane ring opened impurity of Docetaxel is greater than 2.0 and tailing factor for docetaxel and its impurities is less than 1.2 in all the conditions.

### Assay

Assay determinations of injection formulations is best performed by taking total contents of the vial and dissolve entire contents into the suitable diluent followed by estimation of the total amount of drug present in the solution against the standard. The recent publication [12] employs a method wherein a portion of the sample is weighed for sample preparation and total drug content present per vial is calculated by weight extrapolation. The method involves taking weight of a labeled vial before taking the sample, emptying the contents, washing and drying the vial to take the empty weight. This is a not only laborious but also can lead to errors of underestimation or overestimation to a significant extent. The present study describes a much elegant method which requires only thorough cleaning of the vials to ensure transfer of the entire contents into the solution during sample preparation. The drug content is estimated using single point external standard calibration using peak area calculations. USP tailing factor, theoretical plates and relative standard deviation for docetaxel peak is found to be 1.1, 72867, 1.0% respectively.

The precision of assay method is evaluated by assaying the six individual sample preparations. The Mean % assay and Relative standard deviation for % assay was found to be 98.7% and 0.64% respectively. Recovery of docetaxel from spiked placebo was conducted. Samples were prepared in triplicate by mixing placebo with docetaxel API equivalent to about 50%, 100%, and 150% of the assay concentration. The % recovery was found to be between 98.0% to 102.0%. A series of solutions of docetaxel standard, are prepared in the concentration range of 50%, to 150% of the assay concentration. Linearity is established by plotting a graph between concentrations versus peak area. The correlation coefficient is found to be of 0.999. This indicates that the assay method is linear from 50% to 150% of the target assay concentration

**Fig. 6.**
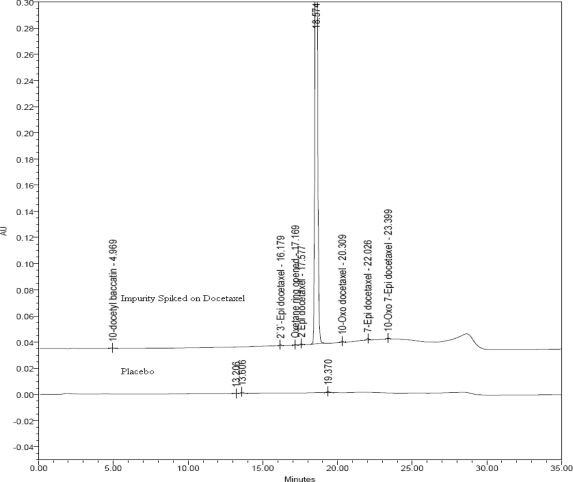
Overlay of Assay chromatograms of placebo (bottom) and impurities spiked on Docetaxel (top) drug product.

The method is proved to be stability indicating by establishing the peak purity of Docetaxel in the forced degradation samples (Table 5). Different batches of formulation products are analyzed and the results are shown in Table 6. The overlay chromatogram of placebo and impurities spiked on Docetaxel drug product in assay method is shown in Figure 6.

## Experimental

### Chemicals, Standards and Impurities

Acetonitrile (HPLC grade, Merck, India), Methanol (HPLC grade, Merck, India), High pure water is from Mill-Q water purification system from Millipore, Acetic acid (AR grade, Merck, India).Docetaxel trihydrate Working standard, Impurity standards of 10-DAB-III-2′,3′-Epidocetaxel, 2′-Epidocetaxel, Oxetane ring opened impurity of Docetaxel, 10-Oxodocetaxel, 7′-Epidocetaxel, 10-Oxo-7-epidocetaxel.

### Samples

Taxotere^®^ 80mg (Docetaxel Injection concentrate from Sanofi Aventis), Docetere™ 20 mg, Docetere™ 80 mg (Docetaxel Injection concentrate from Dr Reddy’s laboratories limited), Different grades of Polysorbate 80 (Vicapol-80 S from Vishwat chemicals, India, Montanox 80 VGPH from SEPPIC, France, Montanox 80 ESSAI from SEPPIC, France, Montanox 80 HX from NOF Corporation, Japan, Super refined^®^ Polysorbate 80 from Croda Inc, USA).

### Equipments

Agilent 1100 series HPLC system equipped with Quaternery pump with an online degasser, auto sampler, thermostatted Column compartment and variable wavelength detector. Waters alliance HPLC system equipped with 2695 separations module and 2489 UV/VIS detector or 2998 Photodiode array detector. Waters Empower1 software is used for data acquisition, data processing in HPLC.

### Chromatographic conditions

A new gradient method is developed for separating process impurities and degradants of docetaxel from placebo peaks proving the method to be stability indicating. The chromatographic method employs a mobile phase-A consisting of a mixture of water, acetonitrile & methanol in the ratio of 50:20:30(v/v) and a mobile phase-B consisting of a mixture of water & acetonitrile in the ratio of 20:80(v/v). The method employs a gradient program listed in Table-7 for impurities analysis and Table 8 for assay analysis using 150 mm x 4.6 mm, YMC Pack ODS-A, 3 μm column maintained at ambient temperature (25±2°C) and the detector set at wavelength of 230 nm. An Injection volume of 25μL is used for impurities analysis and 5μL is used for assay analysis.

### Sample preparations for Assay and Impurities

Contents of the drug product vial is dissolved in 4 ml of diluent (water:acetonitrile: 50:50, v/v) and transferred into a suitable volumetric flask. Each vial is again washed thoroughly with small portions of diluents to remove the drug product completely into the volumetric flask. Then the solution is made up to the volume with diluent and mixed well.

For 20 mg docetaxel injection, total contents of one vial is dissolved and diluted to 25 ml. For 80 mg docetaxel injection, total contents of one vial is dissolved and diluted to 100 ml. This results in a sample solution of 0.8 mg of docetaxel per ml. A placebo (formulation matrix without drug) sample solution is prepared in similar manner for Docetere formulation. Sample solutions of different grades polysorbate 80 are prepared using equivalent weights into the similar volumes. An accurately weighed amount of docetaxel trihydrate is dissolved in diluent to obtain a standard solution having a known concentration of 0.8 mg/ml.

### Specificity and mass balance study

Specificity is the ability of the method to measure the analyte response in the presence of its potential impurities and degradation products [13]. The specificity of the developed LC method for docetaxel was carried out in the presence of its impurities. Sample is subjected to acid hydrolysis, alkaline hydrolysis and oxidation conditions. Sample is also subjected to thermal and photo degradation in dry state. Different stress conditions are followed to achieve degradation. The degraded samples are diluted to get 0.8mg/ml of docetaxel. The total impurities and assay are estimated with respective methods.

### Limit of detection (LOD) and limit of quantification (LOQ)

The LOD and LOQ for impurities and docetaxel are estimated at a signal-to-noise ratio of 3:1 and 10:1, respectively by injecting a series of diluted solutions with known concentration. Precision study was also carried at the LOQ level by injecting six individual preparations of all impurities and docetaxel and calculating the % RSD. Accuracy at LOQ level is evaluated in triplicate for the impurities by spiking the impurities at the estimated LOQ level to test solution.

### Precision

Precision of assay method is evaluated by carrying out six independent assays of test sample of docetaxel drug product against qualified docetaxel standard and calculated the % RSD. The precision of the impurities method is verified by injecting six individual preparations. Docetaxel drug product spiked with 0.3% of known impurities with respect to docetaxel concentration. % RSD for each known impurity is calculated.

### Accuracy

The accuracy of the assay method is evaluated in triplicate at three concentration levels, i.e. 50, 100 and 150% of the assay concentration. The %recovery is calculated against 0.8mg/ml of standard preparation. Recovery experiments were conducted to determine accuracy of impurities method for the quantification of all impurities in drug product. The study was carried out in triplicate at 0.15%, 0.3% and 0.45% of the test concentration.

### Linearity

Linearity test solutions for the assay method are prepared from docetaxel stock solutions at five concentration levels from 50 to 150% of assay analyte concentration (50, 75, 100, 125 and 150%). The peak area versus concentration data is treated by least-squares linear regression analysis. Linearity solutions for the impurities method were prepared by diluting impurity stock solutions to the required concentrations. The solutions are prepared at different concentration levels from LOQ to 0.45%.

### Robustness

To determine the robustness of the developed method, experimental conditions are deliberately altered and the resolution between docetaxel and its impurities and tailing factor for docetaxel and its impurities are recorded. The effect of flow rate is studied at 0.8ml/min and 1.2ml/min and compared with method flow rate of 1.0ml/min. The effect of column temperature is studied at 20°C and 30°C and compared with method column temperature 25°C. The effect of composition of organic solvents is studied by varying acetonitrile and methanol individually by −5 to +5% while other mobile phase components are held constant and compared with method organic solvent composition.

## Conclusion

The present study emerged with suitable impurities and assay methods for evaluation of pharmaceutical quality of docetaxel in docetaxel injection formulations. The impurities method is designed by taking adequate care to separate process related impurities, degradation products, placebo components from each other and also from docetaxel. The method also identifies the retention times of known impurities and ensures their quantification accurately employing relative response factors. A simpler and accurate assay method for determination of drug content in docetaxel formulation is established.

## Figures and Tables

**Tab. 1.** Forced degradation study data of Docetaxel formulation for Impurities method.

	**Stress Study**		**Unstressed**	**Oxidation**	**Thermal**	**Base**	**Acid**	**Peak Purity**

	**Stress Condition**		**NA**	**3% H_2_O_2_ for 12 hrs**	**100°C for 48 hrs**	**2N NaOH for 1 hr**	**2N HCl for 24 hrs**	

**RRT**	**K^1^**	**Name of Impurity**	**% Impurity**					
0.11	4.2	Unknown	ND	1.22	ND	ND	ND	Passes
0.12	4.6	10-Deacetylbaccatin III	ND	1.63	1.14	9.68	ND	Passes
0.15	6.0	Unknown	ND	ND	ND	0.3	ND	Passes
0.17	7.0	Unknown	ND	0.74	0.16	ND	ND	Passes
0.22	9.3	Unknown	ND	0.48	ND	7.19	ND	Passes
0.26	11.2	Unknown	ND	ND	0.2	0.96	ND	Passes
0.70	31.8	Unknown	ND	ND	ND	ND	0.39	Passes
0.88	40.2	Unknown	0.01	ND	0.12	ND	ND	Passes
0.91	41.6	Unknown	0.26	0.16	ND	0.15	0.36	Passes
0.95	43.5	Unknown	0.01	0.04	0.01	0.01	0.04	Passes
0.97	44.4	Unknown	0.03	0.04	1.26	0.01	ND	Passes
0.98	44.9	Unknown	ND	0.09	ND	ND	ND	Passes
1.00	45.8	Docetaxel	99.31	91.39	86.56	43.79	98.81	Passes
1.05	48.2	10-Oxodocetaxel	0.19	0.18	0.07	0.68	0.2	Passes
1.10	50.5	7-Epidocetaxel	0.14	4.03	10.22	34.68	0.17	Passes
1.13	51.9	10-Oxo 7-epidocetaxel	0.04	0.34	0.26	2.52	ND	Passes
Total degradation			0.69	9.06	13.44	56.21	1.19	NA
Mass Balance			99.5	97.8	98.5	96.4	99.5	NA

NA=Not applicable

**Tab. 2.** Relative Retention time (RRT), Limit of Quantification (LOQ), Relative response factor (RRF) and Recovery data of the Docetaxel and its Impurities.

**Name of the Impurity**	**∼RRT**	**K^1^**	**LOD (in %)**	**LOQ (in %)**	**RRF**	**% RSD at LOQ^a^**	**%Recovery at LOQ^b^**
10-Deacetylbaccatin III	0.11	4.2	0.002	0.006	1.53	2.1	99.0
7-Epi-10-deacetyl-baccatin III	0.20	8.3	NA	NA	NA	NA	NA
7-Epi-10-oxo-10-deacetylbaccatin III	0.22	9.2	NA	NA	NA	NA	NA
2′,3′-Epidocetaxel	0.88	39.9	0.003	0.008	0.96	1.9	106.4
2′-Epidocetaxel	0.94	42.7	0.003	0.009	1.02	3.4	96.4
Oxetane ring opened impurity of Docetaxel	0.97	44.1	0.004	0.011	0.73	2.5	94.7
Docetaxel	1.00	45.4	0.003	0.008	1.00	1.5	100.6
10-Oxodocetaxel	1.05	47.8	0.003	0.014	0.91	3.7	108.3
7′-Epidocetaxel	1.11	50.6	0.003	0.011	1.00	2.4	96.1
10-Oxo-7′epidocetaxel	1.14	52.0	0.002	0.006	1.35	2.6	97.1

NE = Not Established due to non-availability of impurity standards; NA= Not applicable;

a Mean for six determinations;

b Mean for three determinations

**Tab. 3.** Results of recovery study:

**Name of the impurity**	**Amount spiked^a^**			**% Recovery^b^**		
	**50%**	**100%**	**150%**	**50%**	**100%**	**150%**
10-Deacetylbaccatin III	0.15	0.3	0.45	99.1	100.2	99.3
2′,3′-Epidocetaxel	0.15	0.3	0.45	100.1	100.5	99.4
2′-Epidocetaxel	0.15	0.3	0.45	98.2	99.5	98.2
Oxetane ring opened impurity of Docetaxel	0.15	0.3	0.45	97.8	98.2	97.5
10-Oxodocetaxel	0.15	0.3	0.45	99.6	99.7	96.7
7′-Epidocetaxel	0.15	0.3	0.45	96.4	95.7	97.5
10-Oxo-7′-epidocetaxel	0.15	0.3	0.45	98.9	97.2	96.5

a Amount of impurities spiked with respect to 0.30% specification level individually to 0.8 mg/ml of docetaxel;

b Mean±%RSD for three determinations.

**Tab. 4.** Batch analysis of Impurities for different samples.

**Name of the Product**			**Docetere™ 80 mg**		**Docetere™ 20 mg**		**Taxotere® 80 mg**

**Batch No.**			**A7469**	**A8252**	**A7486**	**A8331**	**D6D400**

**Months before Expiry**			**9**	**14**	**10**	**16**	**9**

**RRT**	**K^1^**	**Name**	**% Impurity**				
0.119	4.5	10-Deacetylbaccatin III	ND	ND	ND	ND	ND
0.200	8.3	7-Epi-10-deacetylbaccatin III	ND	ND	ND	ND	ND
0.233	9.8	7-Epi-10-oxo-10-deacetylbaccatin III	ND	ND	ND	ND	ND
0.425	18.6	Placebo	NA	NA	NA	NA	NA
0.447	19.8	Placebo	NA	NA	NA	NA	NA
0.827	37.4	Placebo	NA	NA	NA	NA	NA
0.872	39.5	Unknown	0.01	0.09	0.04	0.06	0.04
0.893	40.5	2′,3′-Eepidocetaxel	ND	ND	ND	ND	ND
0.902	40.9	Unknown	0.09	0.11	0.02	0.04	0.00
0.945	42.9	2′-Epidocetaxel	ND	ND	ND	ND	ND
0.967	43.9	Unknown	0.03	0.05	0.03	0.01	0.12
0.972	44.2	Oxetane ring opened impurity of Docetaxel	ND	ND	ND	ND	ND
0.98	44.5	Unknown	0.01	0.01	0.01	0.05	0.01
1.00	45.5	Docetaxel	NA	NA	NA	NA	NA
1.05	47.8	10-Oxodocetaxel	0.19	0.20	0.22	0.12	0.20
1.11	50.6	7′-Epidocetaxel	0.11	0.09	0.11	0.13	0.04
1.14	52.0	10-Oxo-7′-epidocetaxel	0.02	0.03	0.01	0.04	0.00
Total Impurities			0.45	0.45	0.32	0.45	0.41

NA = Not Applicable; ND = Not Detected

**Tab. 5.** Forced degradation study data of Docetaxel formulation for assay method.

**Stress Study**	**Stress condition**	**Peak purity of Docetaxel**
Unstressed	Unstressed	Passes
Oxidation	3% H2O2 for 12 hours	Passes
Thermal	100°C for 48 hours	Passes
Base	2N NaOH for 1 hour	Passes
Acid	2N HCL for 24 hours	Passes

**Tab. 6.** Batch analysis of assay for different samples.

**Name of the Product**	**Batch No**	**Months before Expiry**	**Label claim**	**Docetaxel Content**	**Docetaxel Concentration**

				**mg/vial**	**mg/ml**
Docetere™ 20	A7486	10	20 mg / 0.5 ml	23.8	40.3
Docetere™ 20	A8331	16	20 mg / 0.5 ml	24.0	40.7
Docetere™ 80	A8252	14	80 mg / 2.0 ml	94.5	40.2
Docetere™ 80	A7469	9	80 mg / 2.0 ml	95.4	40.6
Taxotere® 80	D6D400	9	80 mg / 2.0 ml	96.4	40.8

**Tab. 7.** HPLC Gradient program for impurities analysis.

**Time (min)**	**Flow rate (ml/min)**	**% Mobile Phase A**	**% Mobile Phase B**	**Gradient Curve**
0–25	1.0	100	0	Isocratic
25–60	1.0	100→20	0→80	Linear
60–65	1.0	20→100	80→20	Linear
65–75	1.0	100	0	Equilibration

**Tab. 8.** HPLC Gradient Program for assay analysis.

**Time (min)**	**Flow rate (ml/min)**	**% Mobile Phase A**	**% Mobile Phase B**	**Gradient Curve**
0–25	1.0	95→40	5→60	Linear
25–26	1.0	40→95	60→5	Linear
26–35	1.0	95	5	Equilibration
